# Sclerotherapy Failure Salvage by Oesophageal Transection?

**DOI:** 10.1155/1993/46814

**Published:** 1993

**Authors:** D. S. K. Sarin, S. Mehta

**Affiliations:** Gastroenterology G B Pant Hospital New Delhi 110 022 India


### SCLEROTHERAPY FAILURE SALVAGE BY OESOPHAGEAL TRANSECTION?

### ABSTRACT

*McCormick, P.A., Kaye, G.L., Greenslade, L., Cardin, F., Hobbs, K.E.F., McIntyre, N. and Burroughs, A.K. Esophageal staple transection as a salvage procedure after failure of acute injection sclerotherapy. (1992) Hepatology; 3: 403–406.*

**It is not clear which therapy should be used in patients with bleeding esophageal varices that are not controlled by emergency sclerotherapy. This is a high-risk group with reported mortality rates of between 70% and 90%. We report our 7-year experience with staple transection of the esophagus in this patient group. Of 168 patients (280 bleeding episodes) treated with sclerotherapy, 22 had emergency staple transection for failure to control bleeding. Bleeding was controlled in 20 patients (90%), and 10 patients (45%) survived to leave the hospital, including 4 of 10 patients (40%) with Pugh grade C liver disease. We suggest that emergency staple transection is an effective salvage treatment for this high-risk group. (Hepatology, 1992; 15:403–406.)**

### PAPER DISCUSSION

KEY WORDS: Bleeding varices, oesophageal transection

The present article has explored the possibility of using the salvage procedure of oesophageal transection, in a group of patients in whom sclerotherapy has failed and who carry an extremely high risk of mortality.

Emergency sclerotherapy is now a well established procedure for acute variceal bleeding. It is successful in controlling active bleeding in nearly 90% of patients[1,2]. Failure of sclerotherapy is considered, if despite two[3] or three[2] sessions, the bleeding continues. The prognosis remains poor in these patients with mortality rates in excess of 50%[4]. In this group of patients, the possibilities include the use of emergency shunt procedures, devascularization, transjugular intrahepatic portosystemic shunts (TIPS), orthotopic liver transplantation (OLT) or staple transection of the oesophagus. At present, there are no clear guidelines to recommend one procedure over the other. The single stage staple gun transabdominal transection is simple and better tolerated, though the extent of devascularization is less (8-10 cm) than in the two stage, transthoracic approach used in the Sugiura's procedure (15-16 cm). While McCormack *et al.* in the present series from the Royal Free Hospital[5], have recommended this procedure, a careful look at their data is needed. Only 22 of the 40 sclerotherapy failures could be taken up for transection. While primary haemostasis could be achieved in 20 (90%) patients, only 10 (45%) could leave the hospital and of these, 8 rebled (either from transection line erosions, gastric varices or upper GI bleeding) and 4 died during a follow-up of 28 months. Only 2 (9%) patients remained free of bleeding. Overall, merely 6 of the 40 (15%) sclerotherapy failures survived in their group; and only 27% of those who underwent transection. Jenkins and Sheilds have reported even worse results[6]. They took all 15 of their sclerotherapy failures for transection. While initial control of haemorrhage could be achieved in 87%, only 27% patients survived to leave the hospital. Similar dismal results were reported by Durtschi *et al.*[7].

Emergency shunts have been in and out of favor for the control of acute variceal bleeds. Two recent papers have stressed distinct efficacy of these procedures. Henderson *et al.* from Emory University, performed emergency distal splenorenal shunt in 31 patients. The 30 day mortality in this group was 16.7%. But these patients belonged to Child's A and B category. The data from San Diego, from Dr Orloff *et al.* has been more dramatic. 74 (80%) of the 94 Child's C cirrhotic patients left the hospital after emergency portacaval shunt and 72% of them were still alive at the end of one year[8]. The incidence of portosystemic encephalopathy was 18.7%, though only 9% required dietary restrictions. These excellent results were claimed to be due to early (within 8 hours) surgical intervention and abstinence from alcohol in their patients. Similar good results (62% one year survival) has been described by McDermott, Jr. (unpublished data), superior to the survival observed in the devascularization and sclerotherapy groups. These reports are however, exceptions to the vast literature which suggests limited success with emergency shunts. The preliminary results with TIPS procedure are quite encouraging. While the portohepatic gradient could be moderately decreased (though much less than what could be achieved by surgical decompression), and bleeding controlled in a fair proportion; the incidence of encephalopathy, shunt blockage, sepsis and other complications is rather high after TIPS procedure[10]. Cautious optimism suggests that TIPS should be used to buy time before a more definitive procedure like surgical shunt or OLT could be undertaken.

Very encouraging results with OLT have been reported by Iwatsuki *et al.* in patients with Child's C cirrhosis with bleeding esophageal varices. One and five year survival rates of 80% and 71% have been reported; far superior to what has been accomplished with chronic sclerotherapy or selective shunts[10]. Should OLT be recommended for all patients with bleeding varices? The answer has been provided in an important study from Emory, where patients with good liver function (Child's A and B) were subjected to distal splenorenal shunt; with 91% one year survival and patients with end-stage liver disease (Child's C) were treated by OLT, with 74% one year survival. With the constraints on organ availability and phenomenal cost, OLT should only be recommended for patients with advanced liver disease and failure of sclerotherapy.

In summary, the options today for patients with sclerotherapy failure remain, a shunt operation for patients with relatively good liver functions and an OLT for patients with end-stage liver disease. Only if these modalities are not available, then staple transection, TIPS or devascularization need to be considered.

### REFERENCES

1. Terblanche, J., Yakoob, H.I., Bornman, P.C. *et al.* (1981) Acute bleeding varices: a five year prospective evaluation of temponade and sclerotherapy. *Ann.Surg.*, **194**, 521–530
2. Sarin, S.K., Nanda, R., Gaur, S.K. *et al.* (1985) Repeated endoscopic sclerotherapy for active variceal bleeding. *Ann.Surg.*, **202**, 708–711

3. Burroughs, A.K., Hamilton, G., Phillips, A., Mezzanotte, G., McIntyre, N. and Hobbs, K.E.F. (1989) Comparison of sclerotherapy with staple transection of the oesophagus for the emergency control of bleeding from oesophageal varices. *N.Engl.J.Med.*, **321**, 857–862
4. Bornman, P.C., Terblanche, J., Kahn, D., Jonker, M.A., Kirsch, R.E. (1986) Limitations of multiple injection sclerotherapy sessions for acute variceal bleeding. *S.Afr.Med.J.*, **70**, 34–36
5. McCormack, P.A., Kaye, G.L., Greenslade, L., Cardin, F., Hobbs, K.E.F., McIntyre, N. and Burroughs, A.K. (1992) Oesophageal staple transection as a salvage procedure after failure of acute injection. *Hepatology*, **15**, 403–406
6. Jenkins, S.A. and Shields, R. (1989) Variceal hemorrhage after failed injection sclerotherapy: the role of emergency oesophageal transection. *Br.J.Surg.*, **76**, 49–51
7. Durtschi, M.B., Carrico, C.J. and Johansen, K.H. (1985) Oesophageal transection fails to salvage high-risk cirrhotic patients with variceal bleeding. *Am.J.Surg.*, **150**, 18–23
8. Orloff, M.J., Orloff, M.S., Rambotti, M. and Girard, B. (1992) Is portal-systemic shunt worthwhile in Child's Class C cirrhosis? *Ann.Surg.*, **216**, 256–268
9. Sanyal, A., Freedman, A.M., Shiffman, M.L., Luketic, V.A., Purdum, P.P. and Tisnado, J. (1992) Transjugular intrahepatic portosystemic shunt (TIPS) vs sclerotherapy for variceal hemorrhage: results of a randomized prospective controlled trial. *Hepatology*, **16**, 88A
10. Iwatsuki, S. and Starzl, T.E. (1992) Liver transplantation in the management of bleeding oesophageal varices. *Balliere's Clinical Gastroenterology*, **6**, 517–626

D.S.K. Sarin
Professor of Gastroenterology
Dr S. Mehta
Senior Resident
Gastroenterology
G B Pant Hospital
New Delhi, 110 022
India

